# Esophageal Microperforation due to Calcified Mediastinal Lymph Node Leading to Tracheoesophageal Fistula

**DOI:** 10.1155/2016/9747193

**Published:** 2016-06-06

**Authors:** Sankalp Dwivedi, E. Brooke Schrickel, Fayez Siddiqui, John O'Brien, James Kruer

**Affiliations:** ^1^St. Mary Mercy Livonia Hospital, Department of Internal Medicine, Livonia, MI 48154, USA; ^2^St. Mary Mercy Livonia Hospital, Graduate Medical Education, Livonia, MI 48154, USA

## Abstract

A 42-year-old male presented with worsening gastroesophageal reflux disease symptoms and cough. The clinical symptoms during the early course of illness were striking for aspiration pneumonia. He was given a prescription of proton pump inhibitors and antibiotics. Rapid decline in the clinical condition with worsening respiratory status was noted. Worsening symptoms of fever, cough, and chest pain prompted further diagnostic work-up suggesting esophageal microperforation. Esophagogram was found to be suggestive of tracheoesophageal fistula. The tracheoesophageal fistula was due to subcarinal lymph node of nontuberculous origin.

## 1. Introduction

Microperforations of the esophagus are rare and can be a challenge to diagnose and manage. Mediastinal lymphadenopathy as a cause of tracheoesophageal fistula (TEF) resulting in microperforation of the esophagus has rarely been reported in the literature. TEF are mostly congenital and present in the pediatric population. In adults, malignancy and trauma are major causes. We present an unusual case of acquired TEF due to calcified lymph node that led to esophageal microperforation.

## 2. Case Presentation

A 42-year-old male presenting to the emergency department (ED) was experiencing worsening cough and heartburn for the past three months when lying supine at night. He also complained of chest pain and fever. He described the feeling of something being stuck in his lower chest. His family mentioned that he also had developed halitosis. For the nonresolving cough and the new onset chest pain and fever, a chest X-ray was done that was unremarkable. Initial Computed Tomography (CT) angiogram of the chest failed to demonstrate any pathology other than a calcified subcarinal lymph node at the right hilum. He was later discharged on proton pump inhibitors and antibiotics for possible pneumonia.

**Figure 1:**
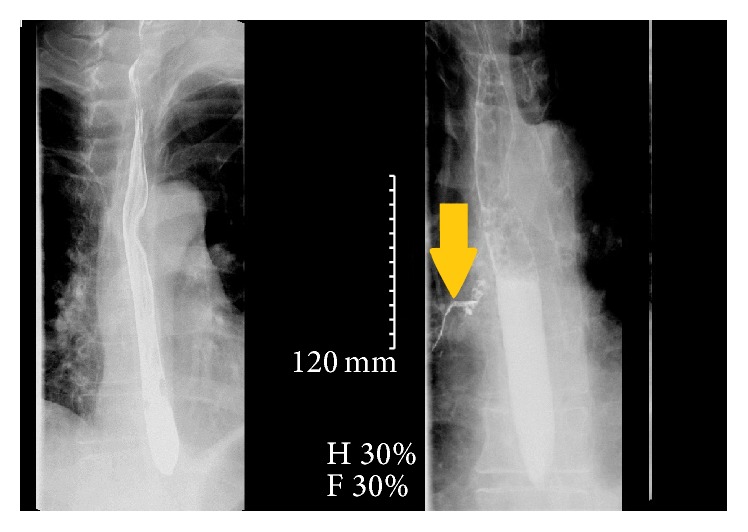
The above images were taken at the end of the study, 3 minutes apart. The image on the right shows the extravasation of contrast into the bronchial tree.

**Figure 2:**
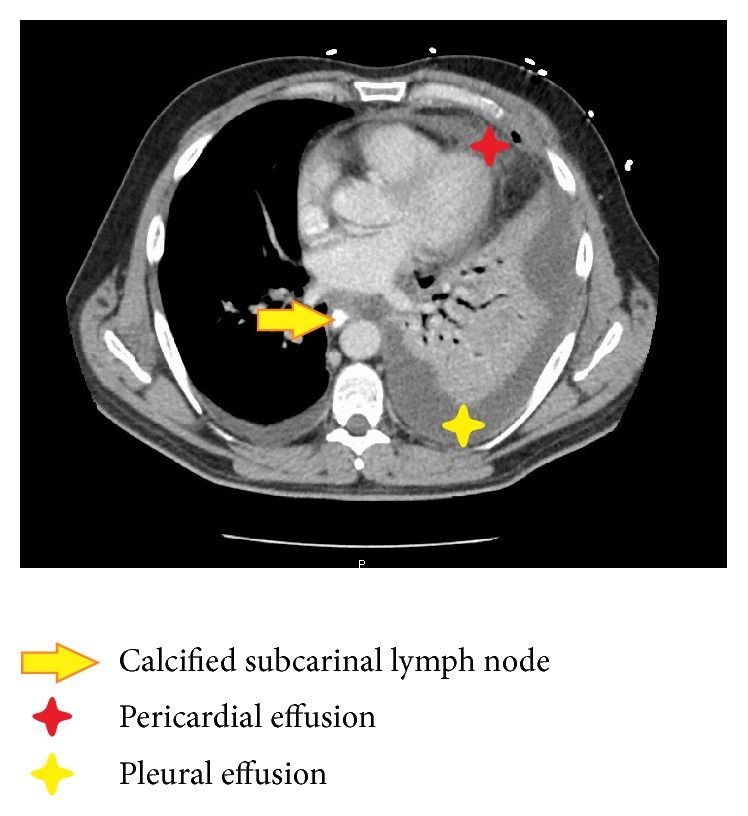
CT chest.

Three days later he presented again to ED with high-grade fever (105°F) and chest pain. Two days later he developed acute hypoxic respiratory failure. Chest X-ray revealed whiteout of the left hemithorax. Ultrasonogram showed evidence of loculated pleural effusion. CT chest with oral contrast revealed large loculated left sided hydropneumothorax containing multiple foci of gas due to empyema, but no frank esophageal perforation was identified. This led to an extensive ICU stay along with multiple laboratory studies to try to identify a systemic infection. The patient received multiple chest tubes and multiple imaging studies to try to identify a cause of his symptoms. It was ultimately a thoracocentesis that revealed a purulent fluid with a pH of less than 6.5 that led us to reexamine a possible gastrointestinal source and repeat the swallow imaging. This time the diatrizoate meglumine and diatrizoate sodium (Gastrografin®, Monroe Township, NJ) swallow was performed and witnessed by the radiologist and gastroenterologist; convincing focal extravasation was identified in the mediastinum only toward the end of the study, suggesting esophageal microperforation of the esophagus (Figure 1). It was thought to be secondary to mediastinal-calcified lymph node that had eroded through the esophagus that was seen on CT chest (Figure 2). Upper endoscopy was attempted to identify any frank perforation, and it was unremarkable. Postcontrast X-rays demonstrated contrast in right medial inferior lung, suggestive of TEF. The patient received a video-assisted thoracoscopic surgery procedure. The surgeons used primary closure along with reinforcement with viable tissue graft wraps from the patient's intercostal muscle. Postoperative pathology report of the pleural fluid and the lymph node did not reveal any obvious pathogens such as tuberculosis or histoplasmosis. The patient's postoperative course was uneventful. The patient responded very well to the procedure and his symptoms finally abated. Patient returned to work one month later and is currently asymptomatic.

## 3. Discussion

Esophageal perforation is a serious condition with mortality rates between 20 and 40% if not identified early [1]. Iatrogenic esophageal perforation is the most common cause of esophageal injury, accounting for 70% of all cases [2], followed by spontaneous perforations (Boerhaave's Syndrome) at 15% [3]. Rarely, mediastinal lymph nodes causing esophageal perforation resulting in bronchoesophageal fistula (BEF) or TEF have also been reported [4–7]. Most reported cases were due to either tuberculous lymph nodes or malignancy [4–7]. To the best of our knowledge, none of the reported cases in literature caused subtle microperforation of the esophagus. In our case, esophageal microperforation was thought to be due to calcified subcarinal lymph node as was seen on CT.

TEF is primarily a congenital condition most commonly seen in the pediatric population; adult cases are rare and predominantly attributed to malignancy, trauma, and infections [4, 8]. In our case, TEF was secondary to esophageal microperforation due to lymphadenopathy. Histoplasma urine antigen was negative and so were the Acid Fast Bacilli (AFB) culture and AFB stain in the pleural fluid. The patient was also found to be HIV negative. Since tuberculosis and histoplasmosis were negative in our patient, presence of granulomatous disease was less likely. No etiology in the biopsy of the lymph node was identified for the calcified lymphadenopathy that led to the esophageal microperforation and TEF. The patient did not have any past medical history to suggest a predisposition to TEF.

Initial CT combined with oral contrast did not suggest frank esophageal leak or esophageal respiratory tract fistula. Careful review of the diatrizoate meglumine and diatrizoate sodium swallow did not identify focal extravasation, but toward the end of the study minimal collection of the contrast in the mediastinum was noticed. Upper EGD was unremarkable to identify any perforation. For this complicated case, it was only after piecing together the relevant clinical findings—subtle nature of presentation, unremarkable EGD, and diatrizoate meglumine and diatrizoate sodium study showing very late minimal extravasation—that esophageal microperforation was diagnosed. To our knowledge there is no reported literature describing this phenomenon; esophageal microperforation is in and of itself a rare clinical entity, and the etiology of calcified lymphadenopathy and associated TEF makes this case even more unique.

This case highlights the challenges related to the diagnosis and management of esophageal microperforation. Neither a CT scan with oral contrast nor an esophagogram may pick up esophageal microperforation; hence, the diagnosis is contingent on appraisal of the entire clinical picture and may not be as dramatic as frank esophageal perforations carrying a high mortality rate. After the patient's second ED visit within three days, he was finally admitted to the hospital. The total duration of his symptoms was approximately 3 weeks before the final diagnosis was made. This case highlights that failure to diagnose microperforation may delay the prompt management of this possibly life threatening condition.
